# Inhibition of *Leishmania infantum* Trypanothione Reductase by New Aminopropanone Derivatives Interacting with the NADPH Binding Site

**DOI:** 10.3390/molecules28010338

**Published:** 2023-01-01

**Authors:** Valentina Noemi Madia, Davide Ialongo, Elisa Patacchini, Cécile Exertier, Lorenzo Antonelli, Gianni Colotti, Antonella Messore, Valeria Tudino, Francesco Saccoliti, Luigi Scipione, Andrea Ilari, Roberta Costi, Roberto Di Santo

**Affiliations:** 1 Istituto Pasteur-Fondazione Cenci Bolognetti, Dipartimento di Chimica e Tecnologie del Farmaco, “Sapienza” Università di Roma, p.le Aldo Moro 5, 00185 Rome, Italy; 2 Istituto di Biologia e Patologia Molecolari del CNR c/o Dipartimento di Scienze Biochimiche, Sapienza Università di Roma, p.le Aldo Moro 5, 00185 Roma, Italy; 3 Dipartimento di Scienze Biochimiche, Sapienza Università di Roma, p.le Aldo Moro 5, 00185 Roma, Italy; 4 D3 PharmaChemistry, Italian Institute of Technology, Via Morego 30, 16163 Genova, Italy

**Keywords:** *Leishmania infantum*, trypanothione reductase inhibition, X-ray crystal structure, NADPH binding site, procaine derivatives, procainamide derivatives, cluster docking

## Abstract

Background: As a result of the paucity of treatment, Leishmaniasis continues to provoke about 60,000 deaths every year worldwide. New molecules are needed, and drug discovery research is oriented toward targeting proteins crucial for parasite survival. Among them, trypanothione reductase (TR) is of remarkable interest owing to its vital role in *Leishmania* species protozoan parasite life. Our previously identified compound **1** is a novel chemotype endowed with a unique mode of TR inhibition thanks to its binding to a formerly unknown but druggable site at the entrance of the NADPH binding cavity, absent in human glutathione reductase (hGR). Methods: We designed and synthesized new 3-amino-1-arylpropan-1-one derivatives structurally related to compound **1** and evaluated their potential inhibition activity on TR from *Leishmania infantum* (LiTR). Cluster docking was performed to assess the binding poses of the compounds. Results: The newly synthesized compounds were screened at a concentration of 100 μM in in vitro assays and all of them proved to be active with residual activity percentages lower than 75%. Conclusions: Compounds **2a** and **2b** were the most potent inhibitors found, suggesting that an additional aromatic ring might be promising for enzymatic inhibition. Further structure–activity relationships are needed to optimize our compounds activity.

## 1. Introduction

Leishmaniasis is a neglected tropical vector-borne disease caused by protozoan parasites from the *Leishmania* species of the family *Kinetoplastidae* that infects numerous mammals, including humans [1]. Human leishmaniasis can result in a spectrum of clinical diseases, dependent upon the infecting species, and consists of three major clinical forms: mucocutaneous leishmaniasis (MCL), which is not self-healing and can potentially be fatal; cutaneous leishmaniasis (CL), which can spontaneously heal, though potentially leaving disfiguring scars; and visceral leishmaniasis (VL), which is fatal if left untreated. MCL often begins with an involvement of the nasal mucosa, including generalized inflammation and ulceration. Ulceration and necrosis of these areas may be severe, resulting in disfigurement and, occasionally, death [2]. CL arises from an infection of epidermal tissue after promastigote host inoculation. In susceptible hosts and immuno-compromised persons, disseminated cutaneous or diffuse cutaneous leishmaniasis may occur as a rare but severe manifestation of CL [3]. VL is caused by *L*. *donovani* in East Africa and the Indian subcontinent, and by *L. infantum* in Europe, North Africa, and Latin America. VL arises from the parasitic infection of phagocytic cells within secondary lymphatic organs, the liver, and bone marrow. The disease is almost always fatal without treatment, with fatality varying according to factors such as age, gender and country of residence. It also contributes significantly to household economic loss, as shown by individual studies across countries [4]. Worldwide, there are an estimated 700,000 to 1,000,000 new cases of Leishmaniases annually. These are widely distributed in tropical and subtropical climate zones and lead to 26,000 to 65,000 deaths. Annual VL estimates are currently less than 100,000 [5].

Currently, antileishmanial treatments (e.g., antimony, amphotericin, paromomycin, miltefosine, aminoquinoline, sitamaquine) are unsatisfactory in terms of safety, efficacy, and pharmacokinetic profiles, representing a neglected area of research and drug discovery, in particular for VL. This diverges with the therapeutic need for VL treatments, the number of affected patients, and the associated fatalities [6], highlighting the need to develop novel therapies to treat *Leishmania* parasite infections. Advances in this field of research suggest that new compounds targeting proteins crucial for parasite survival but absent in the human host represent a promising strategy against leishmaniasis [7]. Among these, trypanothione reductase (TR), a flavoenzyme-reducing trypanothione (TS_2_) in its T(SH)_2_ form, is of remarkable interest [8]. Indeed, unlike the human redox defense machinery, these parasites possess TS_2_ as the main detoxifying system against oxidative damage, allowing them to neutralize hydrogen peroxide produced by macrophages during infection [9]. The human homolog, glutathione reductase (hGR), maintaining glutathione in a reduced state (GSH), has a different substrate binding site in terms of both volume and charge distribution. However, although similarities in structure and function between TR and GR exist, these enzymes recognize specific substrates (trypanothione and glutathione), suggesting the possibility of designing specific and selective inhibitors of the parasite enzyme without off-target activity affecting the host [10]. The crystal structure of *L*. *infantum* TR has been shown to be very similar to the structure of other trypanosomatid TRs, such as those of *T*. *cruzi* and *T*. *brucei*. TR is a two-fold symmetrical homodimer in which each subunit is formed by three domains, the interface domain, the NADPH binding domain, and the FAD binding domain. Reduction in TS_2_ follows a mechanism involving the transfer of two electrons from NADPH via FAD to the Cys52–Cys57 disulfide bridge. TR is a validated drug target in both *Leishmania* and *Trypanosoma* because all the attempts to obtain TR-knockout mutants failed and its downregulation causes strong impairment of infectivity [11]. Moreover, the drugs currently used to treat leishmaniasis target the trypanothione metabolism, confirming the idea of the druggability of this enzyme. It is also worthy of note that the high sequence homology of TRs from different sources (80–100%) make them a valuable target for developing a single, broad-spectrum drug active against all trypanosomatids [12].

**Figure 1:**
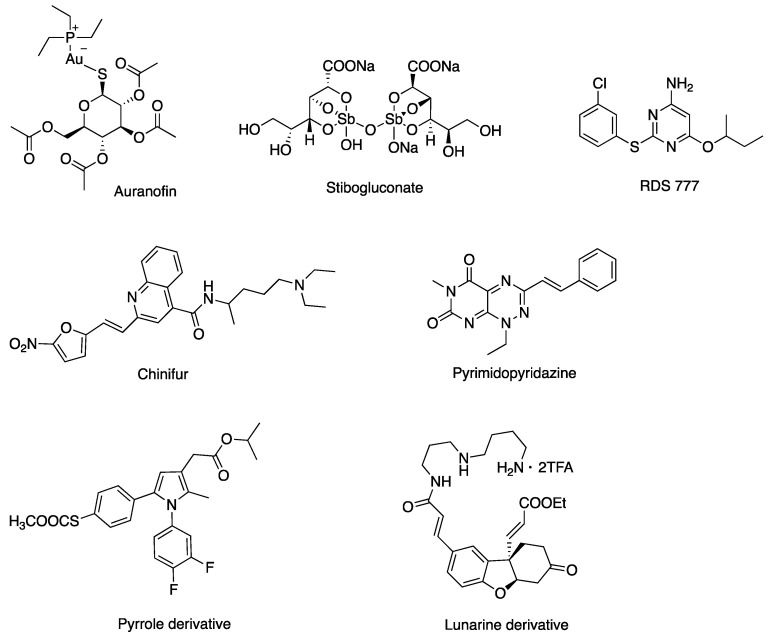
Structures of small TR-inhibiting molecules described in the literature.

Several small molecules have been described for their ability to inhibit TR activity, such as metals and metal-containing compounds (i.e., auranofin), antimony-containing drugs (i.e., stibogluconate), diaryl sulfides (i.e., RDS 777), polyamine analogs (i.e., eflornithine), quinoline-based (i.e., chinifur) and pyrimidopyridazine-based compounds, azole derivatives (such as pyrrole derivatives), and lunarine analogs (Figure 1) [10].

Their inhibition modes include competition with trypanothione due to their binding to the wide TS_2_ cavity (an ability shared by most of the characterized inhibitors) and redox cysteines inactivation due to a metal binding to Cys52 and Cys57 in the catalytic site. However, despite several TR inhibitors having been reported in the literature, none of them have reached the drug development stage. There are several reasons for this, including their often suboptimal potency, their poor selectivity leading to toxicity, and their low bioavailability or biodistribution causing inactivity in animal models. Nonetheless, the large hydrophobic active site of TR makes its inhibition by small molecules challenging [12]. Therefore, the need to discover new scaffolds able to inhibit TR remains urgent.

**Figure 2:**
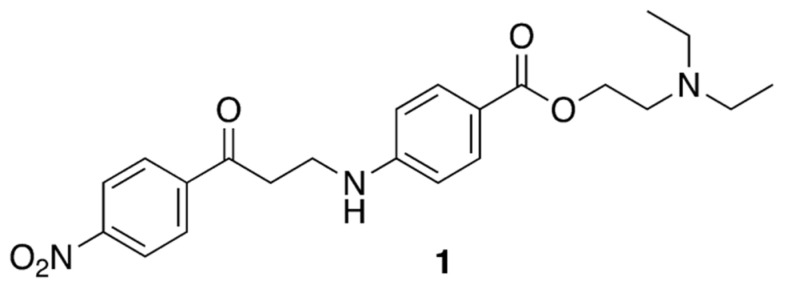
Structure of the previously reported 3-amino-1-phenylpropan-1-one derivative (**1**) active on the *Li*TR NADPH binding site.

Recently, we identified a novel chemotype endowed with a unique mode of TR inhibition [10]. Indeed, compound **1** (2-(diethylamino)ethyl 4-((3-(4-nitrophenyl)-3-oxopropyl)amino)benzoate, Figure 2) proved to be capable of binding to a previously unknown but druggable site at the entrance of the NADPH binding cavity, thus competing with NADPH. Interestingly, the compound **1** binding site identified in the crystal structure is unique, since it is present in TR but not in human homologs such as hGR. This cavity may be considered less appealing for the development of specific TR inhibitors due to the ubiquitous nature of NADPH. Even so, hitting this druggable site in the NADPH cavity represents a promising strategy enabling selectivity over hGR. Moreover, this compound showed an IC_50_ of 7.5 μM against *Li*TR, determined by a luminescence assay, without exerting any activity against hGR (IC_50_ > 85 μM). Further studies showed that compound **1** induced a dose-dependent anti-proliferative effect on *L. infantum* promastigotes with an IC_50_ of 12.44 μM, suggesting that the compound is indeed able to reach its target at the parasite level [10].

**Figure 3:**
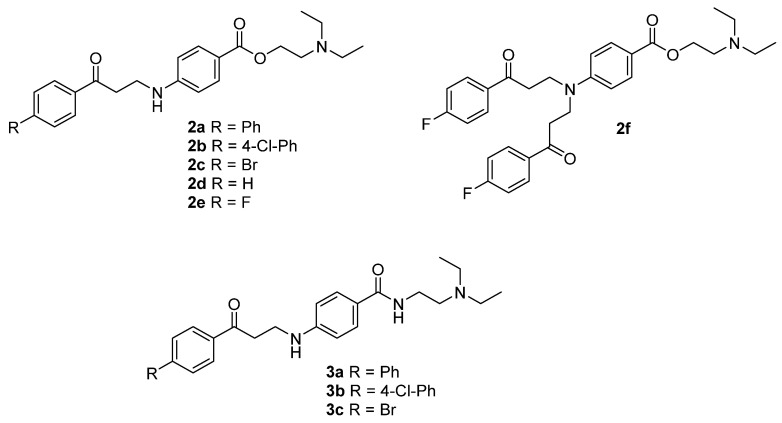
Structures of the newly designed 3-amino-1-arylpropan-1-one derivatives **2a**–**f** and **3a**–**c**.

Taking into account these promising results, we designed and synthesized new 3-amino-1-arylpropan-1-one derivatives and evaluated their activity against *Li*TR. All the newly designed compounds **2a**–**f** and **3a**–**c** (Figure 3) were conceived by carrying out substitutions in position 4 of the acetophenone moiety in order to deepen the role of the nitro group. Indeed, although the nitro group has a long history of use in medicinal chemistry and the nitro compounds are part of an important group of drugs with different pharmacological activities, in particular to treat parasitic infections (especially visceral leishmaniasis, Chagas’ disease, and human African trypanosomiasis [13]), the issues related to nitroaromatic compounds are contradictory, as the nitro group is considered both a pharmacophore, or its integral part, and a structural alarm [14]. Even if the toxicity of nitro compounds depends on their specific chemical structure, the introduction of a nitro group in a molecule modifies the physiochemical and electronic properties and it is often associated with increased mutagenicity and carcinogenicity [15]. Indeed, different studies show the correlation between nitro-substituted small molecule drugs and genotoxicity and DNA damage [16,17,18]. In light of the above, we decided to modify the 4-nitroacetophenone moiety. Specifically, the compounds of series **2** were synthesized by replacing the nitro group with a hydrogen, fluorine, and bromine atom or with a phenyl or 4-chlorophenyl ring, obtaining derivatives **2a**–**e**. The substitution with the hydrogen atom was designed to allow us to investigate the role of the nitro group, and to verify whether its removal could lead to an activity drop. The replacement with halogen atoms (fluorine and bromine), on the other hand, was conceived in order to enable us to look for further interactions through hydrogen/halogen bonds with the amino acid residues present in the binding site. Indeed, as observed in our in silico model, several polar amino acids surround the nitro group of the compound **1** binding site (i.e., Asn254, Arg222). Furthermore, given the presence of the Arg222 adjacent to the binding site, we envisioned that the introduction of an additional aromatic moiety (phenyl and 4-chlorophenyl rings) might lead to a possible formation of a *π*–cation interaction.

Notably, compound **2f** was obtained as a by-product from the nucleophilic aliphatic substitution between the procaine moiety and the 4-fluorophenylpropan-1-one core by derivatizing the NH group. Thanks to the quantifiable amount obtained, we decided to also test this compound as an analogue of **2e** in order to evaluate the effect of an additional phenylpropan-1-one portion on TR activity. Moreover, by applying a similar approach, we also synthesized the corresponding amide derivatives **3a**–**c** in order to evaluate the role of the ester group.

## 2. Results and Discussion

### 2.1. Chemistry

**Scheme 1:**
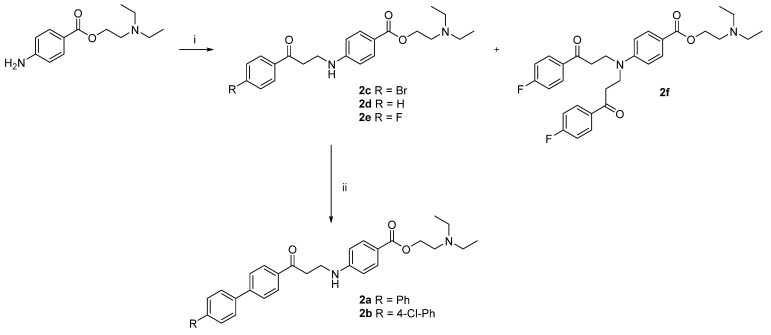
Synthetic route to ester derivatives **2a**–**f**. i: appropriate arylpropanone, Et_3_N, THF *dry*, reflux, 15 h, 25–53% yield; ii: proper arylboronic acid, tetrakis(triphenylphosphine)palladium(0), Na_2_CO_3_ 15% aq, 1,2-dimethoxyethane, reflux, 3–5 h, 60–98% yield (see Materials and Methods section).

The synthesis of the ester derivatives **2a**–**f** is outlined in Scheme 1. Compounds **2c**–**e** were synthesized via an S_N_2 alkylation reaction of the proper commercially available 3-chloro-1-arylpropanone with the commercially available procaine hydrochloride (used as a free base) in dry tetrahydrofuran (THF) using Et_3_N as a base and performing the reaction at reflux for 15 h [19]. Interestingly, the substitution of 3-chloro-1-(4-fluorophenyl)propan-1-one as the reagent in the same conditions gave both the mono- and disubstituted derivatives (**2e** and **2f**, respectively), which were separable by chromatography. Lastly, compounds **2a**,**b** were obtained through a Suzuki–Miyaura cross-coupling reaction [20], starting from the bromo derivative **2c** in the presence of tetrakis(triphenylphosphine)palladium(0) as a catalyst and using the proper arylboronic acid under basic conditions in 1,2-dimethoxyethane at reflux for 3–4 h under an inert atmosphere.

**Scheme 2:**
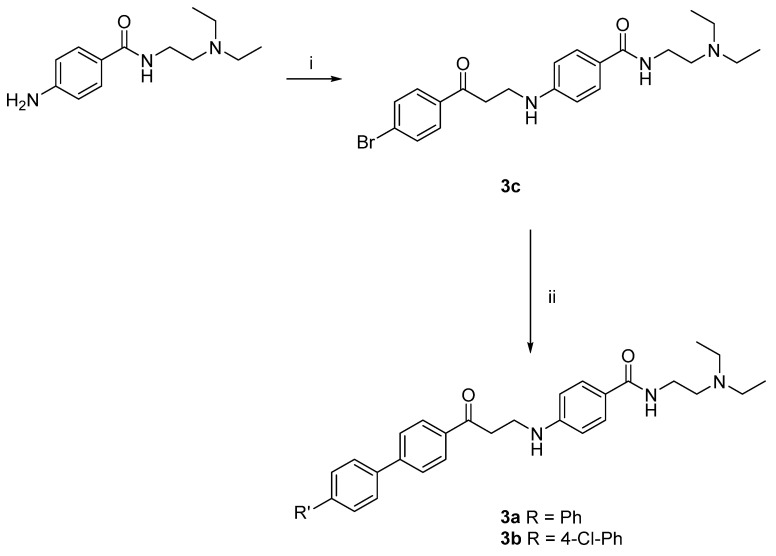
Synthetic route to amide derivatives **3a**–**c**. i: 1-(4-bromophenyl)-3-chloropropan-1-one, Et_3_N, THF *dry*, rt, 15 h, 32% yield; ii: proper arylboronic acid, tetrakis(triphenylphosphine)palladium(0), Na_2_CO_3_, 1,2-dimethoxyethane, reflux, 4–5 h, 48–49% yield (see Materials and Methods section).

The synthesis of the amide derivatives **3a**–**c** was performed as reported in Scheme 2. The synthetic approach resembles the one described above for compounds **2a**–**f**. It is noteworthy that the synthetic pathway starts with a nucleophilic aliphatic substitution reaction of the commercially available procainamide hydrochloride (used as a free base) conducted at room temperature to obtain derivative **3c**.

The detailed synthetic procedures and the analytical and spectroscopic data of the synthesized compounds are reported in the Materials and Methods section.

### 2.2. In Vitro Enzymatic Assay

**Figure 4:**
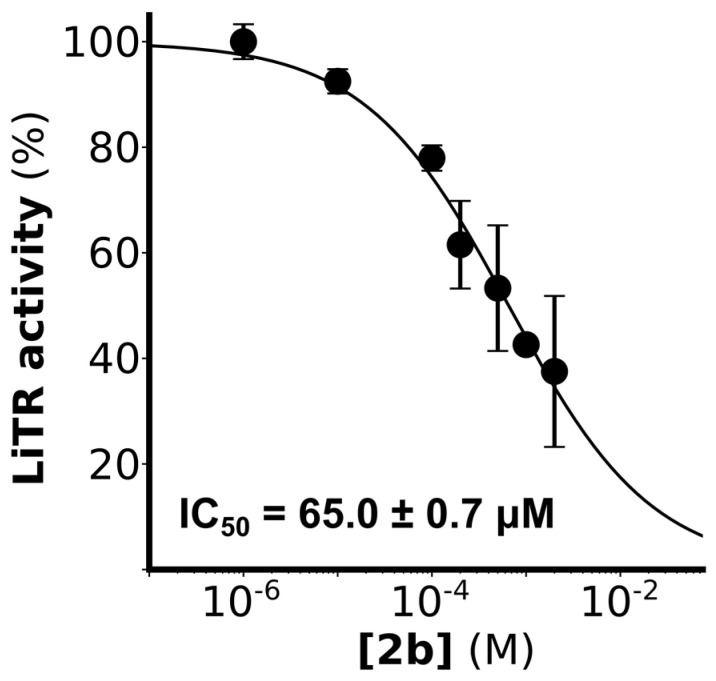
*Li*TR−**2b** inhibition kinetics. The IC_50_ was determined at 25 °C in the presence of 150 μM trypanothione and 100 μM NADPH. Each point represents the average of three experiments.

The newly synthesized compounds **2a**–**f** and **3a**–**c** were tested at a concentration of 100 μM to measure their ability to inhibit *Li*TR enzymatic activity, and all of them proved to be capable of inhibiting TR at the reported conditions (Table 1). Notably, eight out of the nine compounds reduced *Li*TR activity by at least 25%. Furthermore, the best acting compound, namely **2b**, reduced the activity of the enzyme by 47%. Additionally, we determined an IC_50_ of 65.0 μM for this compound at 25 °C (Figure 4). As a comparison, compound **2b** showed a > 150 μM IC_50_ value on the human homolog glutathione reductase (Figure S10). It is worthy of note that the effectiveness of inhibitor **2b**, even though endowed with a lower activity with respect to the parent compound **1**, indicates that the replacement of the nitro group, endowed with several toxicity issues, represents an effective strategy for the development of *Li*TR inhibitors.

Regarding the substituent in the 4-position of the phenylpropan-1-one moiety, it was observed that the best results were obtained with the introduction of a phenyl or 4-chlorophenyl group. Indeed, esters **2a**,**b** and the corresponding amides **3a**,**b** showed lower values of residual activity with respect to the corresponding 4-hydrogen substituted (**2d**) or 4-halogen substituted counterparts (**2c**,**e**,**3c**), suggesting that the introduction of an additional aromatic ring might be a promising approach for enzymatic inhibition. In fact, the activity decreases in the following order for both esters and amides: 4-Cl-Ph > Ph > Br. Regarding the introduction of an additional arylpropan-1-one moiety, no encouraging results were obtained, the disubstituted compound **2f** being less active than the corresponding analogue **2e**.

### 2.3. Docking Experiments

In order to obtain an in silico confirmation of the data arising from the enzymatic assays, we performed molecular docking simulations on all the newly synthesized compounds using the X-ray structure of the enzyme. We performed the docking experiments using AutoDock4 and we selected the TR binding positions on the basis of the RMSD cluster analysis, with an RMSD-tolerance of 2 Å.

We used the X-ray structure of TR in its oxidized form and co-crystallized it with inhibitor **1** (PDB: 6ER5) since this compound is structurally similar to the newly synthesized compounds. We know from previous studies [10] that the binding of this compound causes a significant displacement of two arginine residues (Arg222 and Arg228) that in the NADPH-bound TR are involved in the binding of the adenine ribose phosphate moiety.

To validate the docking protocol, we performed a re-docking on compound **1**. Re-docking was performed using Autodock, setting the parameters to the same values used for compounds **2a**–**f** and **3a**–**c**. The re-docked pose with the lowest energy obtained for compound **1** is reported in the Supplementary Materials section (Figure S1).

According to the enzymatic assays, the most populated cluster (thus indicating the most promising compound) belongs to ligand **2b**, which contains 27/100 poses, as shown in Table 2. The poses (generated by the docking procedure) for each compound **2a,c**–**f, 3a**–**c** with the lowest energy in the most populated cluster are reported in the Supplementary Materials (Figures S2–S9) section. The lowest energy pose in the cluster of **2b** displays a binding energy of −3.62 kcal/mol, as calculated by the suite (= van der Waals energy + H bond energy + desolvatation energy + electrostatic energy + final total internal energy + torsional free energy − unbound system’s energy).

**Figure 5:**
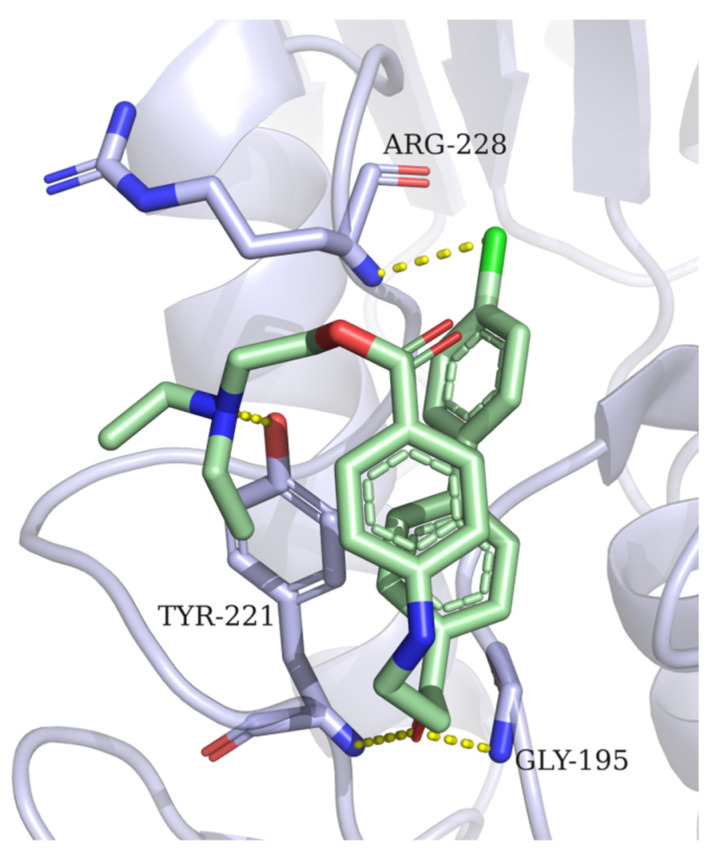
Conformation of compound **2b** with the lowest energy in the most populated cluster, resulting from the docking procedure performed using TR in its oxidized state.

The binding pose of compound **2b** with the lowest energy in the most populated cluster is illustrated in Figure 5. The docking results showed that compound **2b** is bound to the NADPH cavity entrance, forming a weak electrostatic interaction with the aminoacidic residues of Tyr221, Arg228, and Gly195. Specifically, the chlorine atom manages to form a halogen bond with the backbone of the protein (Arg228), while the carbonyl group interacts with the residues Tyr221 and Gly195 via hydrogen bonds. It is interesting to note that the halogen bond of compound **2b** allows its positioning in such a conformation that the two aryl groups can interact via a *π*-stacking interaction, thereby impeding the NADPH entrance. This result may explain the competition between compound **2b** and NADPH observed in vitro.

**Figure 6:**
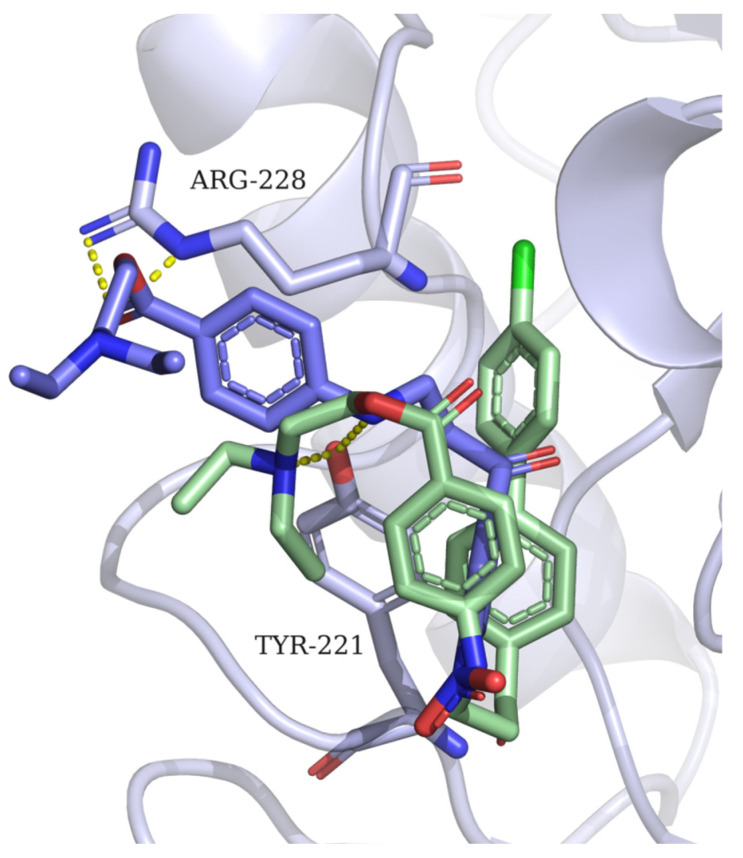
Superposition of inhibitor **1** (in blue), X-ray structure, and compound **2b** (green), modeled by molecular docking.

By comparing the X-ray structure of compound **1** [10] with the conformation of the newly described inhibitor **2b**, generated by docking, we observed that, in both cases, Tyr221 is involved in the ligand–protein interaction by establishing a hydrogen bond with the amino groups (secondary for compound **1** and tertiary for **2b**), as shown in Figure 6.

On the other hand, in our previous work we observed that inhibitor **1** also binds the NADPH cavity entrance through several weak electrostatic interactions with Arg228, Gly197, Asn254, and Arg222, thereby hampering the entrance of the substrate in the binding site [10]. It is relevant to emphasize that the carbonyl group of compound **1** is able to form a hydrogen bond with the guanidine group of Arg228, as shown in Figure 6. This binding mode causes a local conformational change, in which the Arg228 undergoes a 30° rotation (clockwise) relative to the position this residue takes in the TR in its oxidized state. This interaction could play an important role in the inhibition of *Li*TR enzymatic activity. In fact, as we can observe from the docking results, compound **2b** assumes a conformation such that this hydrogen bond is not established. This could explain the lower *Li*TR-inhibiting potency of compound **2b** compared with **1** in enzymatic assays.

## 3. Materials and Methods

### 3.1. Chemistry

#### 3.1.1. General Instrumentation

Melting points were determined on a Bobby Stuart Scientific SMP1 melting point apparatus and are uncorrected. Compound purity was always > 95%, as determined by combustion analysis. The analytical results agreed with the theoretical values to within ± 0.40%. IR spectra were recorded on a PerkinElmer Spectrum-One spectrophotometer. The ^1^H NMR spectra were recorded at 400 MHz on a Bruker AC 400 Ultrashield 10 spectrophotometer (400 MHz). Dimethyl sulfoxide-*d*_6_ 99.9% (CAS 2206-27-1) and Chloroform-*d* 98.8% (CAS 865-49-6) of isotopic purity (Aldrich) were used. Column chromatographies were performed on silica gel (Merck; 70−230 mesh) and on aluminum oxide (Merck; 70–230 mesh). All compounds were routinely checked on TLC by using aluminum-baked silica gel plates (Fluka DC-Alufolien Kieselgel 60 F_254_) or TLC aluminum oxide 60 F_254_ basic (Merck). Developed plates were visualized using UV light. Solvents were reagent grade and, when necessary, were purified and dried using standard methods. The concentration of solutions after reactions and extractions involved the use of a rotary evaporator (Büchi) operating at a reduced pressure (ca. 20 Torr). Organic solutions were dried over anhydrous sodium sulfate (Merck). All solvents were freshly distilled under nitrogen and stored over molecular sieves for at least 3 h prior to use. The analytical results agreed with the theoretical values to within ± 0.40%.

#### 3.1.2. General Experimental Procedures

**General Procedure A (GP-A) for the synthesis of derivatives 2c**–**f and 3c.** Procaine hydrochloride (0.02 mol, 5.45 g) (for the synthesis of compounds **2c**–**f**) or procainamide hydrochloride (0.02 mol, 5.43 g) (for the synthesis of compound **3c**) was solubilized in NaOH 1 N (200 mL) and extracted with ethyl acetate (3 × 100 mL). The organic layer was dried over Na_2_SO_4_ and concentrated under reduced pressure to give a pale yellow oil that was subsequently dissolved in anhydrous THF (90 mL). Triethyl amine (15.7 mL, 0.024 mol) and the proper arylpropan-1-one (0.017 mol) were then added, and the reaction was stirred at reflux for 15 h [21]. The mixture was cooled to ambient temperature and the solvent was reduced under vacuum. Water (300 mL) was added, and the crude product was extracted with ethyl acetate (3 × 200 mL). The organic layers were combined, washed with brine, dried over Na_2_SO_4_, filtered, and concentrated to dryness. The raw material was purified by column chromatography on silica gel (chloroform/methanol 5:1 as eluent) or directly recrystallized (isopropanol) to give pure compound **3c** as a pale yellow solid. For each derivative, the amount of the proper arylpropan-1-one, yield (%), melting point, recrystallization solvent, IR, ^1^H NMR, and elemental analysis are reported.

**General Procedure B (GP-B) for the synthesis of derivatives 2a,b** and **3a,b.** The appropriate arylboronic acid (0.0087 mol) and tetrakis(triphenylphosphine)palladium(0) (1.0 g) were added to a stirred, degassed mixture of the appropriate 3-amino-1-arylpropan-1-one derivatives **2c**–**f** or **3c** (0.0072 mol) in dimethoxyethane (50 mL) and 15% aqueous sodium carbonate solution (50 mL) under argon. The reaction mixture was stirred under reflux for 3–5 h. After cooling at room temperature, water (50 mL) and ethyl acetate (50 mL) were added, and the organic material was extracted into ethyl acetate (2 × 50 mL). The combined organic layers were washed with saturated aqueous sodium chloride solution (2 × 50 mL), dried over Na_2_SO_4_, filtered, and concentrated under reduced pressure. The crude product was purified by column chromatography on silica gel (chloroform/methanol 10:1 for derivatives **2a**,**b** and chloroform/methanol 7:3 for derivatives **3a**,**b** as eluent) [22]. For each derivative, the amount of the proper 3-amino-1-arylpropan-1-one, the amount of the proper arylboronic acid, reaction time, yield (%), melting point, recrystallization solvent, IR, ^1^H NMR, and elemental analysis are reported.

#### 3.1.3. Characterization

**2-(Diethylamino)ethyl 4-((3-([1,1’-biphenyl]-4-yl)-3-oxopropyl)amino)benzoate (2a).** Compound **2a** was synthesized according to GP-B starting from **2c** (1 g); phenylboronic acid (0.32 g); 4 h; 60% yield as a white solid; isopropyl ether; IR ν 3382 (NH), 1696 (CO), 1605 (COO) cm-1; ^1^H NMR (CDCl3) *δ* 1.07 (t, 6H, J = 7.3 Hz, N-CH_2_-*CH_3_*), 2.64 (q, 4H, J = 7.6 Hz, N-*CH_2_*-CH_3_), 2.85 (t, 2H, J = 6.3 Hz, CH_2_-*CH_2_*-N), 3.32 (t, 2H, J = 8 Hz, CO-*CH_2_*-CH_2_), 3.70 (q, 2H, J = 8 Hz, CO-CH_2_-*CH_2_*), 4.35 (t, 2H, J = 7.9 Hz, *CH_2_*-CH_2_-N), 4.64 (t, 1H, J = 6 Hz, NH), 6.59 (d, 2H, J = 8 Hz, benzene C_3′’_-C_5′’_), 7.41 (m, 1H, J = 2.4 Hz, benzene C_4_), 7.47 (t, 2H, J = 8 Hz, benzene C_3_-C_5_), 7.61 (d, 2H, J = 8.6 Hz, benzene C_2_-C_6_), 7.68 (d, 2H, J = 8 Hz, benzene C_3′_-C_5′_), 7.85 (d, 2H, J = 8.6 Hz, benzene C_2′’_-C_6′’_), 8.02 (d, 2H, J = 8 Hz, benzene C_2′_-C_6′_), Anal. Calcd for C_28_H_32_N_2_O_3_: C, 75.65; H, 7.26; N, 6.30%. Found: C, 75.95; H, 7.28; N, 6.31%.

**2-(Diethylamino)ethyl 4-((3-(4’-chloro-[1,1’-biphenyl]-4-yl)-3-oxopropyl)amino)benzoate (2b).** Compound **2b** was synthesized according to GP-B starting from **2c** (1 g); 4-chlorophenylboronic acid (0.42 g); 3 h; 98% yield as a white solid; isopropyl ether; IR ν 3378 (NH), 1694 (CO), 1606 (COO) cm-1; ^1^H NMR (CDCl_3_) *δ* 1.07 (t, 6H, J = 7.3 Hz, N-CH_2_-*CH_3_*), 2.65 (q, 4H, J = 7.6 Hz, N-*CH_2_*-CH_3_), 2.84 (t, 2H, J = 6.3 Hz, CH_2_-*CH_2_*-N), 3.31 (t, 2H, J = 8 Hz, CO-*CH_2_*-CH_2_), 3.70 (q, 2H, J = 6.9 Hz, CO-CH_2_-*CH_2_*), 4.34 (t, 2H, J = 7.9 Hz, *CH_2_*-CH_2_-N), 4.62 (t, 1H, J = 6 Hz, NH), 6.59 (d, 2H, J = 8 Hz, benzene C_3′’_-C_5′’_), 7.43 (t, 2H, J = 8 Hz, benzene C_3_-C_5_), 7.52 (d, 2H, J = 8 Hz, benzene C_2_-C_6_), 7.65 (d, 2H, J = 8 Hz, benzene C_3′_-C_5′_), 7.87 (d, 2H, J = 7.9 Hz, benzene C_2′’_-C_6′’_), 8.01 (d, 2H, J = 8 Hz, benzene C_2′_-C_6′_), Anal. Calcd for C_28_H_31_ClN_2_O_3_: C, 70.21; H, 6.52; Cl, 7.40; N, 5.85; %. Found: C, 70.49; H, 6.51; Cl, 7.41; N, 5.85%.

**2-(Diethylamino)ethyl 4-((3-(4-bromophenyl)-3-oxopropyl)amino)benzoate (2c).** Compound **2c** was synthesized according to GP-A starting from 1-(4-bromophenyl)-3-chloropropan-1-one (2.5 g); 43% yield as a white solid; isopropanol; IR ν 3372 (NH), 1680 (CO), 1599 (COO) cm-1; ^1^H NMR (CDCl_3_) *δ* 1.06 (t, 6H, J = 7.3 Hz, N-CH_2_-*CH_3_*), 2.63 (q, 4H, J = 8 Hz, N-*CH_2_*-CH_3_), 2.82 (t, 2H, J = 6.3 Hz, CH_2_-*CH_2_*-N), 3.24 (t, 2H, J = 6.2 Hz, CO-*CH_2_*-CH_2_), 3.66 (q, 2H, J = 6.1 Hz, CO-CH_2_-*CH_2_*), 4.33 (t, 2H, J = 7.9 Hz, *CH_2_*-CH_2_-N), 4.59 (t, 1H, J = 6 Hz, NH), 6.58 (d, 2H, J = 8 Hz, benzene C_3′_-C_5′_), 7.52 (t, 2H, J = 8 Hz, benzene C_3_-C_5_), 7.72 (d, 2H, J = 8 Hz, benzene C_2_-C_6_), 7.87 (d, 2H, J = 8.6 Hz, benzene C_2′_-C_6′_), Anal. Calcd for C_22_H_27_BrN_2_O_3_: C, 59.07; H, 6.08; Br, 17.86; N, 6.26 %. Found: C, 59.12; H, 6.10; Br, 17.85; N, 6.27 %.

**2-(Diethylamino)ethyl 4-((3-oxo-3-phenylpropyl)amino)benzoate (2d).** Compound **2d** was synthesized according to GP-A starting from 3-chloro-1-phenylpropan-1-one (2 g); 53% yield as a white solid; isopropanol; IR ν 3374 (NH), 1675 (CO), 1598 (COO) cm^−1^; ^1^H NMR (CDCl_3_) *δ* 1.07 (t, 6H, J = 7.1 Hz, N-CH_2_-*CH_3_*), 2.63 (q, 4H, J = 7.1 Hz, N-*CH_2_*-CH_3_), 2.84 (t, 2H, J = 6.3 Hz, CH2-*CH_2_*-N), 3.30 (t, 2H, J = 5.9 Hz, CO-CH_2_-*CH_2_*), 3.68 (q, 2H, J = 6.1 Hz, CO-*CH_2_*-CH_2_), 4.33 (t, 2H, J = 6.3 Hz, *CH_2_*-CH_2_-N), 4.62 (t, 1H, J = 6.1 Hz, NH), 6.58 (d, 2H, J = 8.7 Hz, benzene C_3′_-C_5′_), 7.47 (t, 2H, J = 7.6 Hz, benzene C_3_-C_5_), 7.58 (t, 1H, J = 7.4 Hz, benzene C_4_), 7.86 (d, 2H, J = 8.6 Hz, benzene C_2′_-C_6′_), 7.95 (d, 2H, J = 7.4 Hz, benzene C_2_-C_6_), Anal. Calcd for C_22_H_28_N_2_O_3_: C, 71.71; H, 7.66; N, 7.60%. Found: C, 71.55; H, 7.63; N, 7.62%.

**2-(Diethylamino)ethyl 4-((3-(4-fluorophenyl)-3-oxopropyl)amino)benzoate (2e).** Compound **2e** was synthesized according to GP-A starting from 3-chloro-1-(4-fluorophenyl)propan-1-one (2.4 g); 35% yield as a white solid; isopropanol; IR ν 3368 (NH), 1697 (CO), 1669 (COO) cm-1; ^1^H NMR (CDCl_3_) *δ* 1.09 (t, 6H, J = 7.1 Hz, N-CH_2_-*CH_3_*), 2.68 (q, 4H, J = 8 Hz, N-*CH_2_*-CH_3_), 2.87 (t, 2H, J = 6.3 Hz, CH_2_-*CH_2_*-N), 3.26 (t, 2H, J = 6.2 Hz, CO-*CH_2_*-CH_2_), 3.68 (q, 2H, J = 6.1 Hz, CO-CH_2_-*CH_2_*), 4.37 (t, 2H, J = 8 Hz, *CH_2_*-CH_2_-N), 4.65 (t, 1H, J = 6.1 Hz, NH), 6.58 (d, 2H, J = 8.7 Hz, benzene C_3′_-C_5′_), 7.13 (t, 2H, J = 7.6 Hz, benzene C_3_-C_5_), 7.87 (d, 2H, J = 8.6 Hz, benzene C_2′_-C_6′_), 7.95 (m, 2H, J = 8 Hz, benzene C_2_-C_6_), Anal. Calcd for C_22_H_27_FN_2_O_3_: C, 68.37; H, 7.04; F, 4.92; N, 7.25%. Found: C, 68.92; H, 7.02; F, 4.93; N, 7.22%.

**2-(Diethylamino)ethyl 4-(bis(3-(4-fluorophenyl)-3-oxopropyl)amino)benzoate (2f).** Compound **2f** was synthesized according to GP-A starting from 3-chloro-1-(4-fluorophenyl)propan-1-one (2.4 g); 25% yield as a yellow oil; IR ν 1685 (CO), 1597 (CO), 1101.51 (COO) cm^−1^; ^1^H NMR (CDCl_3_) *δ* 1.09 (t, 6H, J = 8 Hz, N-CH_2_-*CH_3_*), 2.67 (q, 4H, J = 8 Hz, N-*CH_2_*-CH_3_), 2.87 (t, 2H, J = 8 Hz, CH2-*CH_2_*-N), 3.26 (t, 4H, J = 7 Hz, CO-*CH_2_*-CH_2_), 3.90 (t, 4H, J = 7 Hz, CO-CH_2_-*CH_2_*), 4.37 (t, 2H, J = 8 Hz, *CH_2_*-CH_2_-N), 6.67 (d, 2H, J = 8 Hz, benzene C_3′_-C_5′_), 7.12 (t, 2H, J = 8 Hz, benzene C_3_-C_5_), 7.95 (t, 2H, J = 8 Hz, benzene C_2_-C_6_), 7.90 (t, 2H, J = 8 Hz, benzene C_2′_-C_6′_), Anal. Calcd for C_31_H_34_F_2_N_2_O_4_: C, 69.39; H, 6.39; F, 7.08; N, 5.22%. Found: C, 69.62; H, 6.38; F, 7.11; N, 5.22%.

**4-((3-([1,1’-Biphenyl]-4-yl)-3-oxopropyl)amino)-N-(2-(diethylamino)ethyl)benzamide (3a).** Compound **3a** was synthesized according to GP-B starting from **3c** (1 g); phenylboronic acid (0.32 g); 5 h; 49% yield as a yellow solid; isopropyl ether; IR ν 3362 (NH), 1665 (CO), 1611 (COO) cm-1; ^1^H NMR (DMSO d_6_) *δ* 0.95 (t, 6H, J = 7.3 Hz, N-CH_2_-*CH_3_*), 2.59 (q, 4H, J = 7.9 Hz, N-*CH_2_*-CH_3_), 2.75 (t, 2H, J = 6 Hz, CH_2_-*CH_2_*-N), 3.56 (t, 2H, J = 7.5 Hz, CO-*CH_2_*-CH_2_), 3.70 (q, 2H, J = 7.5 Hz, CO-CH_2_-*CH_2_*), 4.35 (t, 2H, J = 7.9 Hz, *CH_2_*-CH_2_-N), 6.22 (t, 1H, J = 6 Hz, NH), 6.58 (d, 2H, J = 8 Hz, benzene C_3′’_-C_5′’_), 7.45 (t, 1H, J = 6 Hz, benzene C_4_), 7.51 (t, 2H, J = 7.5 Hz, benzene C_3_-C_5_), 7.61 (d, 2H, J = 8.6 Hz, benzene C_2_-C_6_), 7.76 (d, 2H, J = 8 Hz, benzene C_3′_-C_5′_), 7.85 (d, 2H, J = 8.6 Hz, benzene C_2′’_-C_6′’_), 7.91 (t, 1H, J = 8 Hz, amide NH), 8.22 (d, 2H, J = 8 Hz, benzene C_2′_-C_6′_), Anal. Calcd for C_28_H_33_N_3_O_2_: C, 75.81; H, 7.50; N, 9.47%. Found: C, 75.51; H, 7.49; N, 9.48%.

**4-((3-(4’-Chloro-[1,1’-biphenyl]-4-yl)-3-oxopropyl)amino)-*N*-(2-(diethylamino)ethyl)benzamide (3b).** Compound **3b** was synthesized according to GP-B starting from **3c** (1 g); 4-chlorophenylboronic acid (0.42 g); 4 h; 48% yield as a yellow solid; isopropyl ether; IR ν 3377 (NH), 1607 (CO), 1509 (COO) cm^−1^; ^1^H NMR (DMSO d_6_) *δ* 0.95 (t, 6H, J = 7.3 Hz, N-CH_2_-*CH_3_*), 2.48 (q, 4H, J = 7.9 Hz, N-*CH_2_*-CH_3_), 3.24 (t, 2H, J = 6 Hz, CH_2_-*CH_2_*-N), 3.44 (t, 2H, J = 7.5 Hz, CO-*CH_2_*-CH_2_), 3.70 (q, 2H, J = 7.5 Hz, CO-CH_2_-*CH_2_*), 4.35 (t, 2H, J = 7.9 Hz, *CH_2_*-CH_2_-N), 6.19 (t, 1H, J = 6 Hz, NH), 6.58 (d, 2H, J = 8 Hz, benzene C_3′’_-C_5′’_), 7.56 (t, 2H, J = 7.5 Hz, benzene C_3_-C_5_), 7.60 (d, 2H, J = 8.6 Hz, benzene C_2_-C_6_), 7.81 (d, 2H, J = 8 Hz, benzene C_3′_-C_5′_), 7.84 (d, 2H, J = 8.6 Hz, benzene C_2′’_-C_6′’_), 7.91 (t, 1H, J = 8 Hz, amide NH), 8.08 (d, 2H, J = 8 Hz, benzene C_2′_-C_6′_), Anal. Calcd for C_28_H_32_ClN_3_O_2_: C, 70.35; H, 6.75; Cl, 7.42; N, 8.79%. Found: C, 70.31; H, 6.78; Cl, 7.45; N, 8.75%.

**4-((3-(4-Bromophenyl)-3-oxopropyl)amino)-N-(2-(diethylamino)ethyl)benzamide (3c).** Compound **3c** was synthesized according to GP-A starting from 1-(4-bromophenyl)-3-chloropropan-1-one, (2.3 g); 32% yield as a white solid; isopropanol; IR ν 3352 (NH), 1675 (CO), 1512 (COO) cm^−1^; ^1^H NMR (CDCl_3_) *δ* 1.43 (t, 6H, J = 9 Hz, N-CH_2_-*CH_3_*), 3.02 (q, 4H, J = 9 Hz, N-*CH_2_*-CH_3_), 3.12 (t, 2H, J = 7 Hz, CH_2_-*CH_2_*-N), 3.23 (t, 2H, J = 6.2 Hz, CO-*CH_2_*-CH_2_), 3.63 (q, 2H, J = 6.1 Hz, CO-CH_2_-*CH_2_*), 3.71 (t, 2H, J = 7 Hz, *CH_2_*-CH_2_-N), 4.51 (t, 1H, J = 3 Hz, NH), 6.60 (d, 2H, J = 8 Hz, benzene C_3′_-C_5′_), 7.61 (d, 2H, J = 8 Hz, benzene C_3_-C_5_), 7.71 (d, 2H, J = 8 Hz, benzene C_2_-C_6_), 7.87 (d, 2H, J = 8.6 Hz, benzene C_2′_-C_6′_) 8.18 (br s, 1H, amide NH), Anal. Calcd for C_22_H_28_BrN_3_O_2_: C, 59.20; H, 6.32; Br, 17.90; N, 9.41%. Found: C, 59.19; H, 6.29; Br, 17.95; N, 9.44%.

### 3.2. Biological Assays

#### 3.2.1. Protein Expression and Purification

LiTR has been expressed and purified as reported previously [23].

#### 3.2.2. Enzymatic Assay

Enzymatic inhibition assays were performed at 25 °C using a JASCO V650 spectrophotometer equipped with a JASCO EHC-716 Peltier unit. *Li*TR (50 nM) was mixed with 100 μM of the compounds and 150 μM trypanothione in 50 mM HEPES pH 7.4, 40 mM NaCl, and incubated at 25 °C for 3 min. The reaction was started upon addition of 100 μM NADPH. Oxidation of the NADPH was followed as a decrease in absorbance at 340 nm. The best inhibitor candidate (**2b**) was then tested under the same conditions at concentrations ranging from 0.1 to 200 μM to determine the IC_50_. *Li*TR activity was determined as V_0_(compound)/V_0_(control)*100, where V_0_ is the initial velocity. The IC_50_ was determined using a dose response logistic equation defined by the Kaleidagraph software as ymin + (ymax-ymin)/(1 + (x/IC_50_)^slope).

#### 3.2.3. Docking Studies

The AutoDock4 docking package [24] was used for ligand flexible docking simulations. The structure of TR in oxidized form downloaded from the protein data bank (PDB code: 6ER5) was set up as the receptor for the docking protocol. The TR structure was edited using software from the ADT package to remove all water molecules, add hydrogen atoms, and add Kollman charges. Nonpolar hydrogens and lone pairs were then merged into each atom within the macromolecule and a Gasteiger partial charge was assigned to each ligand. The pdbqt files were generated with ADT for the protein and the ligands (pdb files containing the partial charge and the autodock atom type). A grid box of 30 × 40 × 28 points, with a spacing of 0.392 Å, was positioned at the active site gorge, centered at 6511 × −33,921 × 16,357 Å with respect to the origin. The Lamarckian genetic algorithm (LGA) was employed to run the docking job for each ligand with 100 runs and a RMSD-tolerance of 2 Å. The maximum number of generations was 27,000 and the maximum number energy evaluations was 2,500,000.

## 4. Conclusions

Within the family of trypanosomatids, there are various species of the *Leishmania* genus responsible for different forms of leishmaniasis. The recent interest from pharmaceutical companies and from international communities in leishmaniasis has increased as the flows of migration and climate changes have allowed the further expansion of these diseases in Europe and in the United States of America. There are currently few available therapies, and these are long lasting and accompanied by various side effects. In addition, the situation appears to be aggravated by the emergence of drug-resistant strains of *Leishmania*. It is evident that there is a need to identify new targets and new molecules potentially applicable in therapeutic settings.

One of the most promising targets appears to be the enzyme TR, which is characteristic of trypanosomatids and essential for their survival. It allows parasites to maintain a reducing environment within the cell, protecting it from the oxidative stress produced by the host’s immune system. The structural differences of TR in terms of size and charge of the active site, compared with its human homologue glutathione reductase, allow selective inhibitors to be developed, thereby reducing adverse effects.

Recent studies have highlighted the ability of compound **1** to selectively inhibit TR, while crystallography has shown that this molecule interacts with the enzyme in a formerly unreported binding pocket. Whereas previously synthesized inhibitors carried out their activity by competing with the substrate in the active site, compound **1** binds at the entrance of the cavity intended for NADPH, thus preventing the transfer of two electrons from the NADPH and reducing trypanothione disulfide to trypanothione dithiol.

Taking into account these encouraging results, we designed a new series of compounds structurally related to **1**, retaining its 3-amino-1-arylpropan-1-one core. We synthesized two series of derivatives characterized either by the ester group or the amide group, and by different substituents in the 4-position of the acetophenone moiety. Enzyme inhibition assays performed on the *Li*TR showed that the best results were obtained for derivatives endowed with a 4-chlorophenyl or phenyl group in position 4 of the acetophenone moiety, with ester derivatives endowed with higher potencies with respect to the amide derivatives. Indeed, the best inhibitor was **2b**, with a residual *Li*TR activity of 47% and an IC_50_ value of 65.0 μM. Consequently, the ester function and the presence of the 4-chlorophenyl substituent appear to be the most promising characteristics for the synthesis of new 3-amino-1-aryl propanone derivatives for use as new TR inhibitors. Even though further investigation will be needed to study the structure–activity relationships of this new class of TR inhibitors, the experimental results described herein confirmed our hypothesis, according to which a manipulation on the nitro group is possible. Moreover, compound **2b** showed a more than two-fold higher IC_50_ value, indicating that it has a good selectivity vs. TR and suggesting that it may not target host cells. Therefore, inhibitor **2b**, retaining an inhibitory activity against *Li*TR in the micromolar range, even though to a lesser extent than the parent compound **1** (as also observed *in silico*), proved that the replacement of the nitro group, endowed with intrinsic contradictions and considered a structural alarm, represents a viable and effective approach for the development of *Li*TR inhibitors with improved safety and characterized by a novel binding mode.

## Figures and Tables

**Table 1:** Enzymatic activities against *Li*TR of the newly synthesized compounds **2a–f** and **3a–c** in comparison with reference compound **1**.

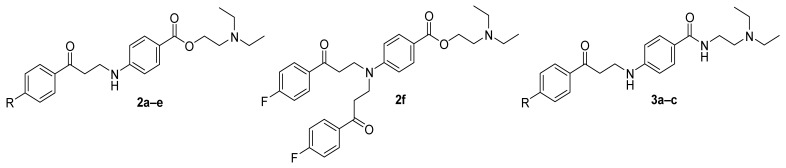		
Cpd	R	*Li*TR Activity (% at 100 µM) ^1^
**2a**	Ph	55.4 ± 13.3
**2b**	4-Cl-Ph	47.0 ± 9.7
**2c**	Br	73.8 ± 4.6
**2d**	H	73.8 ± 4.4
**2e**	F	60.5 ± 4.1
**2f**	-	75.0 ± 2.4
**3a**	Ph	68.4 ± 6.7
**3b**	4-Cl-Ph	59.4 ± 2.4
**3c**	Br	87.3 ± 1.9
**1**	-	<10.0 [10]

^1^ Percentage of *Li*TR activity in presence of 100 μM of inhibitors.

**Table 2:** Most populated clusters and corresponding lowest binding energies of the newly synthesized compounds **2a**–**f** and **3a**–**c**.

Name	Lowest Binding Energy	Number of Conformations in Cluster
**2a**	−3.65 kcal/mol	17
**2b**	−3.62 kcal/mol	27
**2c**	−3.61 kcal/mol	16
**2d**	−3.50 kcal/mol	14
**2e**	−2.91 kcal/mol	13
**2f**	−3.49 kcal/mol	5
**3a**	−3.45 kcal/mol	23
**3b**	−3.59 kcal/mol	24
**3c**	−3.93 kcal/mol	25

## Data Availability

The data presented in this study are available on request from the corresponding author.

## Supplementary Materials

The following are available online at https://www.mdpi.com/article/10.3390/molecules28010338/s1, Figure S1: Pose of compound **1**, generated by the re-docking procedure; Figures S2–S9: Conformations of compounds **2a,2c–f**, **3a–c** with the lowest energy in the most populated cluster; Figure S10: Inhibition kinetics of the glutathione reductase in the presence of compound **2b**.

## Sample Availability

Samples of the compounds **2a–f** and **3a–c** are available from the authors.
