# Supplementary material for: Inhibition of Leishmania infantum Trypanothione Reductase by New Aminopropanone Derivatives Interacting with the NADPH Binding Site
Source: Molecules. 2023 Jan 1;28(1):338. doi: 10.3390/molecules28010338 (PMC9823735; doi:10.3390/molecules28010338)
Supplement: Supplementary file 1 [file molecules-28-00338-s001.zip › molecules-2064581-supplementary.pdf]

Inhibition of *Leishmania infantum* Trypanothione Reductase by New Aminopropanone Derivatives Interacting with the NADPH Binding Site

Valentina Noemi Madia [1], Davide Ialongo [1], Elisa Patacchini [1], Cécile Exertier [2], Lorenzo Antonelli [2,3], Gianni Colotti [2], Antonella Messore [1], Valeria Tudino [1], Francesco Saccoliti [4], Luigi Scipione [1], Andrea Ilari [2,*], Roberta Costi [1,*] and Roberto Di Santo [1]

[1] Istituto Pasteur-Fondazione Cenci Bolognetti, Dipartimento di Chimica e Tecnologie del Farmaco, "Sapienza" Università di Roma, p.le Aldo Moro 5, I-00185 Rome, Italy; valentinanoemi.madia@uniroma1.it (V.N.M.); davide.ialongo@uniroma1.it (D.I.); elisa.patacchini@uniroma1.it (E.P.); antonella.messore@uniroma1.it (A.M.); valeria.tudino@gmail.com (V.T.); luigi.scipione@uniroma1.it (L.S.); roberta.costi@uniroma1.it (R.C.); roberto.disanto@uniroma1.it (R.D.S.)

[2] Istituto di Biologia e Patologia Molecolari del CNR c/o Dipartimento di Scienze Biochimiche, Sapienza Università di Roma, p.le Aldo Moro 5, Roma, Italy; andrea.ilari@cnr.it (A.I.); Cécile Exertier cecile.exertier@uniroma1.it (C. E.); gianni.colotti@cnr.it (G.C.)

[3] Dipartimento di Scienze Biochimiche, Sapienza Università di Roma, p.le Aldo Moro 5, Roma

[4] D3 PharmaChemistry, Italian Institute of Technology, Via Morego 30, 16163, Genova (Italy); francesco.saccoliti@iit.it (F.S.)

Contents

Figure S1: Conformations of compound **1**.

Figure S2: Conformation of compound **2a**;

Figure S3: Conformation of compound **2c**;

Figure S4: Conformation of compound **2d**;

Figure S5: Conformation of compound **2e**;

Figure S6: Conformation of compound **2f**;

Figure S7: Conformation of compound **3a**;

Figure S8: Conformation of compound **3b**;

Figure S9: Conformation of compound **3c**.

Figure S10: Inhibition kinetics of the glutathione reductase in the presence of compound **2b**.

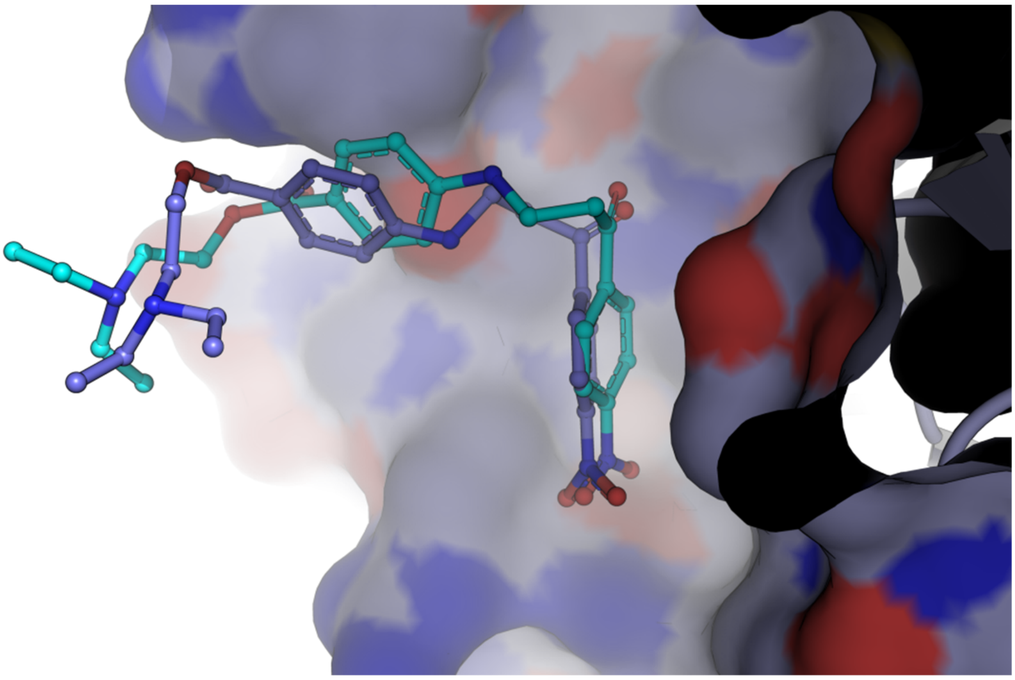

Figure S1: Superposition of crystal structure of compound **1** (medium blue) with the conformation generated by re-docking procedure for the same ligand (light blue).

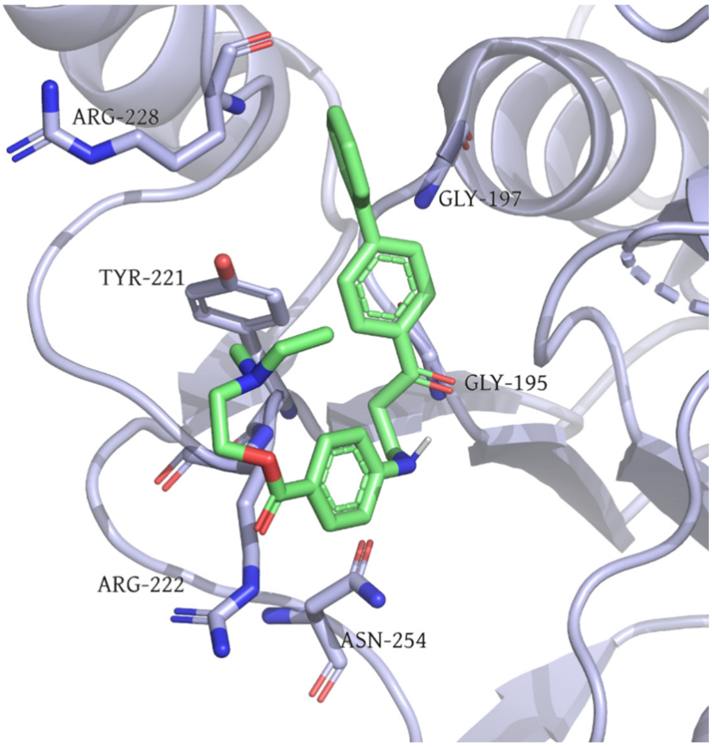

Figure S2: Conformation of compound **2a** with the lowest energy in the most populated cluster.

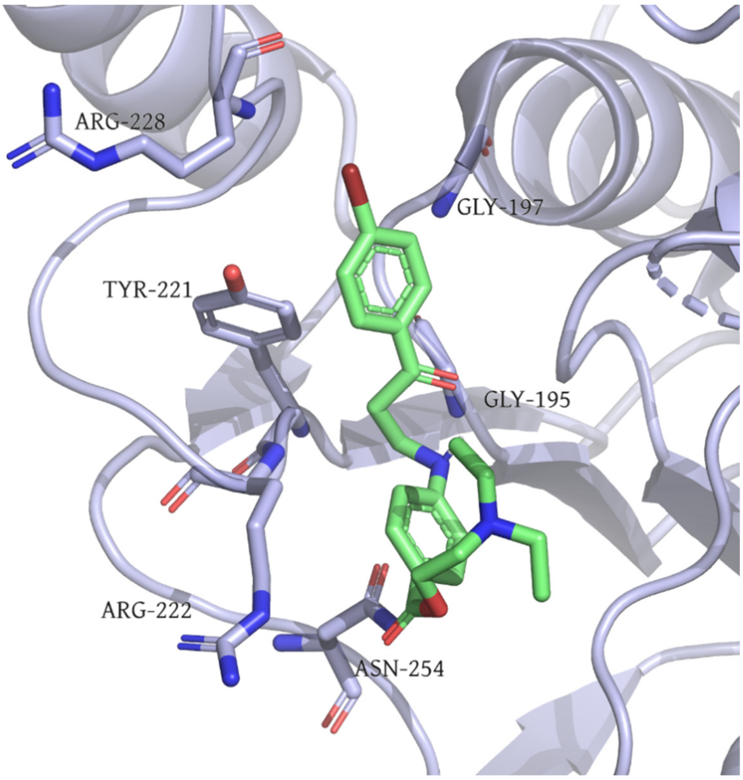

Figure S3: Conformation of compound **2c** with the lowest energy in the most populated cluster.

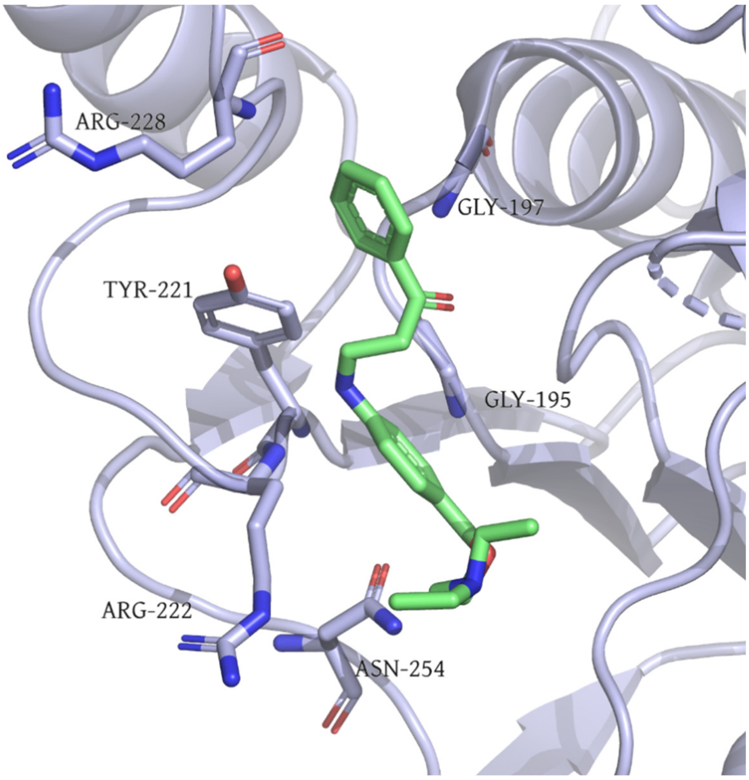

Figure S4: Conformation of compound **2d** with the lowest energy in the most populated cluster.

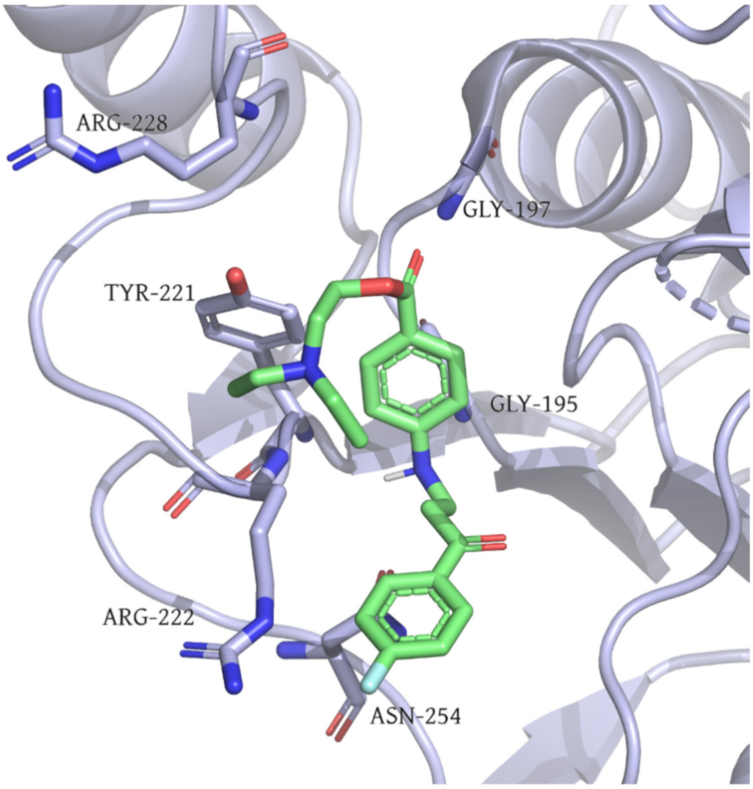

Figure S5: Conformation of compound **2e** with the lowest energy in the most populated cluster.

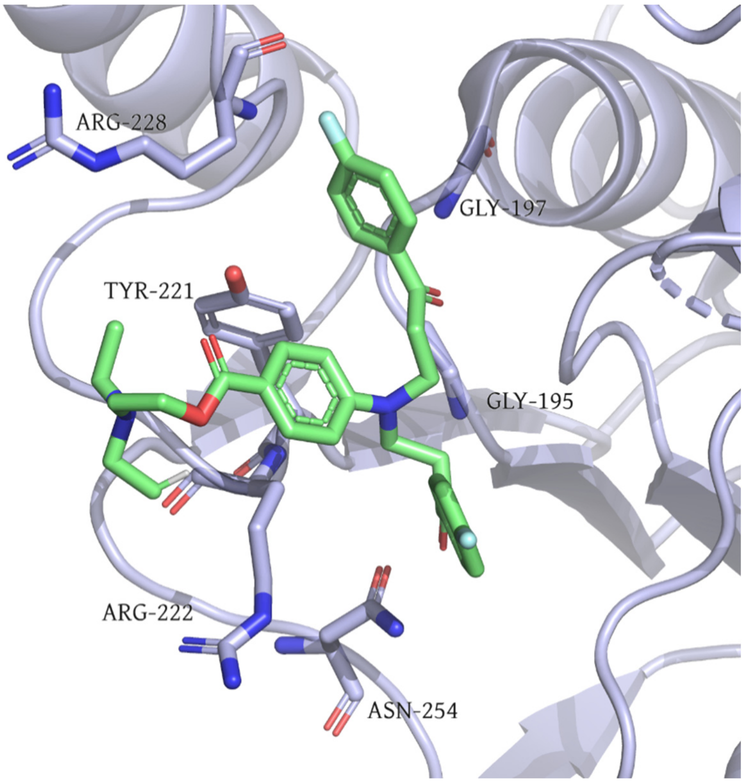

Figure S6: Conformation of compound **2f** with the lowest energy in the most populated cluster.

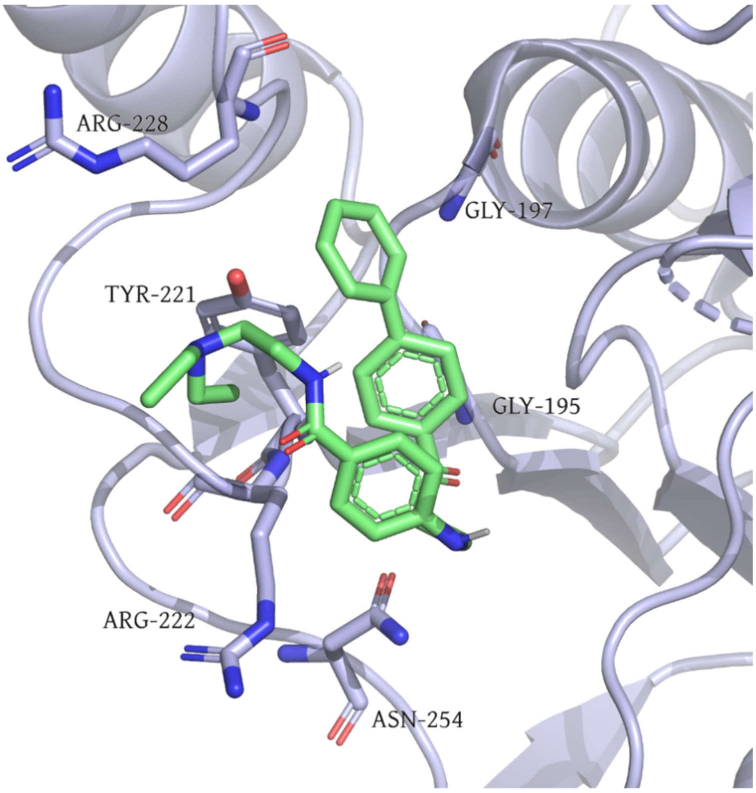

Figure S7: Conformation of compound **3a** with the lowest energy in the most populated cluster.

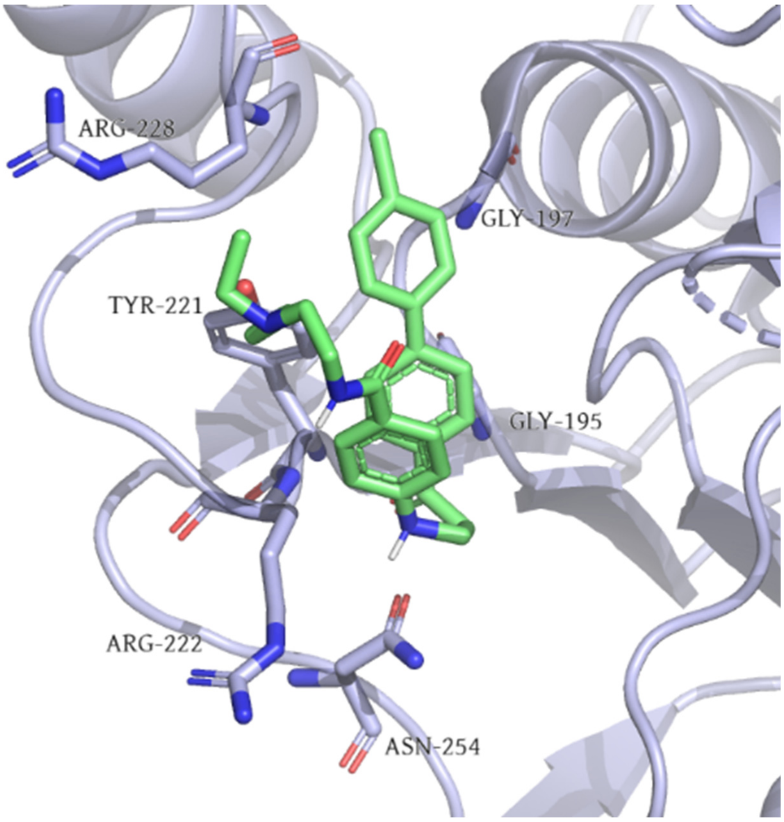

Figure S8: Conformation of compound **3b** with the lowest energy in the most populated cluster.

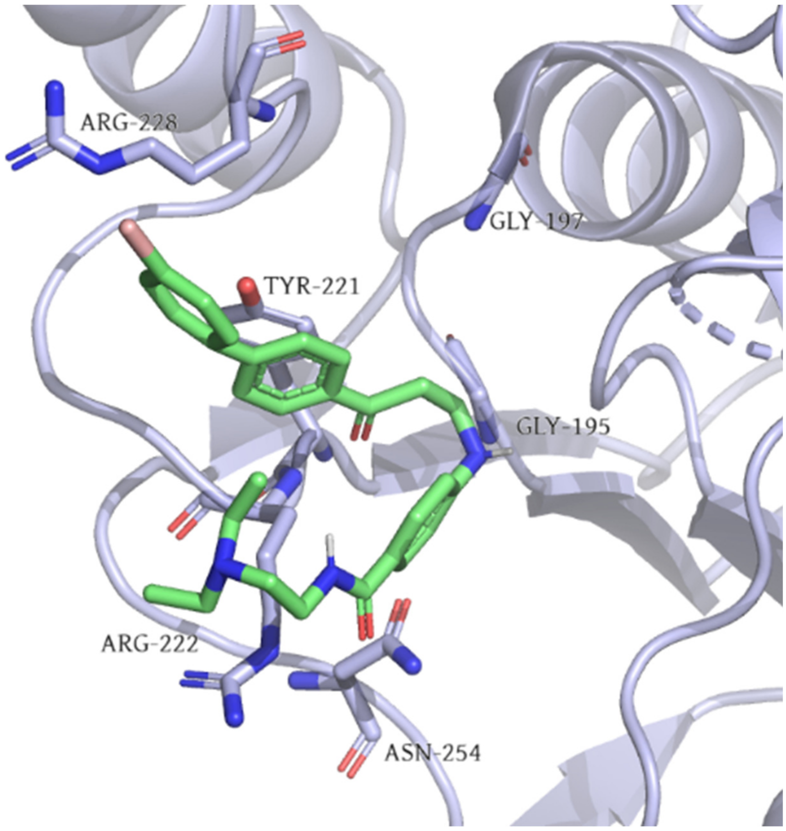

Figure S9: Conformation of compound **3c** with the lowest energy in the most populated cluster.

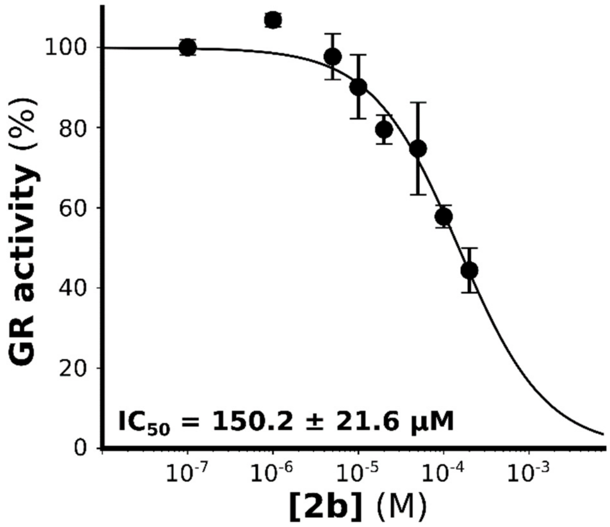

Figure S10. Inhibition kinetics of the glutathione reductase in the presence of compound 2b. Inhibition assays were performed at 25 °C using a JASCO V650 spectrophotometer equipped with a JASCO EHC-716 Peltier unit. Glutathione Reductase (GR) 50 nM, (purchased from Sigma Aldrich) was mixed with varying concentrations of compound 2b (0.1 to 200 μM) and 150 μM oxidized glutathione in 50 mM HEPES pH 7.4, 40 mM NaCl; and incubated at 25°C for 3 minutes. The reaction was started upon addition of 100 μM NADPH. Oxidation of NADPH was followed as a decrease in absorbance at 340 nm. GR activity was determined as V0(compound)/V0(control)*100 where V0 is the initial velocity. The IC50 was determined using a dose response logistic equation defined by the Kaleidagraph software as ymin+(ymax-ymin)/(1+(x/IC50)^slope).
